# Social connection in long-term care homes: a qualitative study of barriers and facilitators

**DOI:** 10.1186/s12877-024-05454-8

**Published:** 2024-10-22

**Authors:** Hannah Chapman, Jennifer Bethell, Neha Dewan, Madalena P. Liougas, Gill Livingston, Katherine S. McGilton, Andrew Sommerlad

**Affiliations:** 1 Division of Psychiatry, University College London, 6th Floor, Maple House, 149 Tottenham Court Road, London, W1T 7NF UK; 2 KITE Research Institute, Toronto Rehabilitation Institute – University Health Network, Toronto, Canada; 3 Institute of Health Policy, Management and Evaluation, University of Toronto, Toronto, Canada; 4 Department of Physical Therapy Education, College of Health Sciences, Western University of Health Sciences, Lebanon, OR USA; 5 Rehabilitation Sciences Institute, Temerty Faculty of Medicine, University of Toronto, Toronto, Canada; 6 Camden and Islington NHS Foundation Trust, London, UK; 7 Lawrence S. Bloomberg, Faculty of Nursing, University of Toronto, Toronto, Canada

**Keywords:** Social connection, Social engagement, Social connectedness, Loneliness, Long-term care home, Nursing home

## Abstract

**Background:**

Social connection is a basic human need and is essential to quality of life. It is associated with better mental and physical health outcomes for long-term care (LTC) home residents and is a key aspect of quality of care and person-centred care. There are considerations for LTC homes that may present obstacles to and opportunities for social connection. It is therefore important to understand what restricts or enables good social connection in LTC homes, to guide better quality care and future interventions in this population. This qualitative study aims to identify barriers and facilitators to social connection for LTC residents.

**Methods:**

We used thematic analysis to describe themes derived from individual and group qualitative interviews from 67 participants (18 residents, 17 staff members and clinicians, 32 family members and friends) recruited from LTC homes in the United Kingdom and Canada.

**Results:**

Themes were grouped into four categories: (1) becoming familiar with life in the LTC home to support social connection; (2) physical and virtual access beyond the LTC home as strategies to maintain contact; (3) getting to know residents to deepen relationships; (4) person-centred approaches to build social connection. ‘Becoming familiar with life in the LTC home to support social connection’ described the benefits of counteracting the institutionalized feel of LTC homes, enabling LTC residents to spend time in meaningful ways, and increasing freedom of mobility around the home. ‘Physical and virtual access beyond the LTC home as strategies to maintain contact’ related to the benefits of outings, providing support with technology, and involving family and friends in LTC home life. ‘Getting to know residents to deepen relationships’ related to the benefits of using routine care and interactions as opportunities for social contact, using family and friend knowledge as a resource, and fostering resident relationships. ‘Person-centred approaches to build social connection’ included considering physical, mental, cognitive, and sensory impairments, accounting for adjustment and sociability, using communal spaces well, and prioritizing psychosocial needs.

**Conclusions:**

This study identifies barriers and facilitators to social connection for LTC residents which can be addressed in care policies, staff selection and training, and can inform policies and interventions to build and maintain social connection in LTC homes.

**Clinical trial number:**

clinicaltrials.gov ID NCT05315960.

**Supplementary Information:**

The online version contains supplementary material available at 10.1186/s12877-024-05454-8.

## Introduction

Social connection describes how individuals relate to each other, encompassing both observable aspects such as engagement with social networks and the experiences of loneliness or social connectedness [1]. Social connection is a human need, essential to quality of life [2] and associated with improved mental and physical health, including for long-term care (LTC) home residents [1, 3]. It is also a key aspect of quality of care and person-centred care in LTC homes [4]. LTC residents have challenges forming and maintaining relationships compared to those in the community, as they live in communal settings but are separated from previous social networks [5]. In addition, many have cognitive, sensory or mobility impairments, which can increase their risk of social isolation due to difficulty maintaining conversation, communicating with or recognising others, and challenges participating in activities [6].

Previous literature has explored aspects of LTC that negatively impacts residents’ capacity to form and maintain relationships. Some residents find it difficult to accept life in LTC, particularly if they lacked choice on living there [7], feel ‘cut off’ from the outside world and lack belonging [8], experience a loss of personal identity [9], and feel out of place with other residents [7]. In addition, a lack of common interests may hinder interactions and prevent close resident relationships [10], as does a lack of opportunities for social engagement [6], and negative expectations around loneliness with age [11]. Connections between LTC residents can also be hindered by negative perceptions of the cognitive ability of others, creating communication difficulties and subsequent social divide between residents with and without dementia [8]. Conversely, connections with family members and outside communities may enable residents’ social connection and can be achieved through visits and contact with people outside [10] and activities. LTC residents can feel more comfortable living in an LTC home if they have trusting relationships with staff caring for them [12], which may be particularly important for LTC residents lacking outside relationships [13].

Homes often attempt to provide socialization through leisure activities [14], particularly for those who have recently moved to LTC homes [15]. However, there is little evidence on the effectiveness of interventions, with a systematic review finding only 10 studies on group interventions (seven reminiscence and three other therapeutic groups) which increased social connection [16]. Other studies focus on specific aspects of social connection such as loneliness [17]. It is therefore valuable to explore individual experiences of how social connection is enabled or prevented, and how specific interventions or policies can help. Although limited, existing evidence finds psychosocial interventions, such as reminiscence groups or support groups, to increase scores of social- and health-related measures, suggesting a positive impact on the social wellbeing of LTC residents [16]. LTC residents, staff members and clinicians, and family and friends of residents offer unique perspectives which may be useful to explore in combination. Previous studies combining these perspectives did not investigate social connection but related constructs such as quality of life [18] or personal relationships [19].

We therefore aim to understand the perspectives of residents, their family and friends, staff and clinicians on the barriers and facilitators to social connection in LTC homes, taking a broad view of both home-level and individual-level factors to explore how they might impact social connection for LTC residents.

## Methods

### Study design

This qualitative study was conducted as part of the SONNET study (social connection in long-term care home residents), which aims to develop a new measure of social connection in LTC residents. We conducted the study through a post-positivist lens, whereby an objective reality may exist, but we acknowledge that this is likely to be influenced by our own experiences and values [20]. Ethical approval was granted by the NHS Health Research Authority (22/LO/0145) and the University Health Network in Canada (21-5976).

### Setting and sample

LTC residents with and without dementia, LTC staff members and clinicians, and family and friends of residents, were purposively sampled in the UK and Canada. LTC residents were included if they were aged 65 or over and had mental capacity to give informed consent at the time of data collection and were excluded if they had active severe mental illness. Researchers recruiting participants were trained to assess mental capacity in line with UK (www.legislation.gov.uk/ukpga/2005/9) or Ontario (www.ontario.ca/page/mental-capacity) Mental Capacity legislation, so ensured that participants understood the information relevant to the study and could weigh the risks and benefits to make a decision before obtaining written consent. There was no eligibility restriction on the duration the LTC residents had lived in the home. Staff members and clinicians were included if they worked at an LTC home over the past two years. Family and friends of LTC residents were included if they visited the resident at least monthly.

### Recruitment

In the UK, all participant groups were recruited from care home teams from three NHS trusts (Camden and Islington NHS Foundation Trust, Oxford Health NHS Foundation Trust, and Northumbria Healthcare NHS Foundation Trust), email newsletters from the national care home research network ENRICH, and LTC homes directly, where LTC home managers helped to identify eligible residents and staff members. Study posters were also sent out widely to advertise the study, where interested participants could contact the study team directly to express interest. Canadian participants were recruited through networks and organizations representing LTC residents and staff, and families across Ontario, Canada, as well as through individually operated and chain LTC homes. Interested participants were given the opportunity to ask questions and time to decide whether to take part before a meeting online or in person was arranged to obtain informed consent and conduct the interview.

### Data collection

The semi-structured interviews were conducted between May 2022 and June 2023, and guided by interview guides for each participant type (appendix 1a-c) based on previous study findings and our research questions. Interview questions explored aspects of social connection since moving into an LTC care home, including the quality of residents’ social life, how it has changed over time, and what influences building or maintaining social connection in this environment. Interviews comprised individual interviews for all participant types, in addition to dyadic interviews for LTC residents and families, between staff members, and one focus group for a subset of staff members. Interviews were conducted by a member of the research team (AS, HC, MM, or two Northumbria NHS Foundation Trust research nurses in the UK, and JB and ES in Canada), and co-facilitated by a second researcher in some instances. In the UK, all LTC resident interviews were conducted in-person in LTC homes, and staff and clinician interviews were conducted virtually using the video-calling platform Microsoft Teams. Family and friend interviews were conducted both in-person and virtually. In Canada, interviews were conducted either in-person in LTC homes (for some resident and family interviews) and virtually for all other participants.

After collecting sociodemographic data using a structured data collection form, interviews took 30–60 min and were recorded using Microsoft Teams or an audio-recorder, and then transcribed verbatim and anonymized by the research team. Each participant received a gift voucher (£20 in the UK and $35 in Canada) as compensation. We stopped recruiting and interviewing participants once we judged that we had reached data saturation for each participant group, meaning that no new themes emerged during our data analysis [21].

### Data analysis

We selected a subsample of five interview transcripts representing different views and experiences, including interview from each key collaborator group, to develop a coding framework [22]. The codebook was guided by the data and pre-defined research objectives and described both objective and experienced aspects of living in LTC homes. All five members of the research team (AS, HC, JB, ML, ND) each reviewed the selected interview transcripts to extract new codes. Previous research suggests that aspects of social connection, such as loneliness, are similarly experienced in LTC homes globally [23]. We therefore planned to combine Canadian and UK data for our analysis, though we considered during the analysis if there were differences in responses between the two countries.

All members of the research team coded data, with one researcher per interview transcript, using Dedoose v9.0.17 (2021, www.dedoose.com). Four previously unread interviews were randomly selected (including Canadian and UK transcripts for LTC resident, staff, and family and friend interviews) and all coders each independently applied the codebook. Coding agreement was quantified using Dedoose and codes where agreement was less than moderate (Cohen’s kappa < = 0.60) were discussed, and the codebook was updated to resolve ambiguities. Once double-coding of four transcripts indicated moderate to high inter-rater reliability (Cohen’s kappa > 0.60), one researcher (HC) coded each subsequent transcript [24]. We analyzed the data by thematic analysis, following Braun and Clarke’s 6-step approach, whereby after the research team (AS, HC, JB, ML, ND) (1) became familiar with the data and (2) generated codes, the primary author (HC) (3) combined these codes into themes, (4) continuously reviewed these themes, before (5) determining the significance of themes and (6) reporting findings [25].

We created and regularly refined mind maps throughout the above steps 3 to 5 of the thematic analysis to explore connections between and link themes, and continuously develop their conceptualization. Theme identification was driven by existing codes and used deductive reasoning to identify themes in line with prior research and existing theory [26].

Given the post-positivist lens applied [20], we used reflective journaling throughout analysis to reduce bias in relation to pre-existing epistemological perceptions. To ensure rigor, we used the COREQ checklist to comprehensively report our study methods in Appendix 3 [27].

## Results

We interviewed 67 participants: 18 LTC residents, 32 family or friends of residents, and 17 LTC staff or clinicians (30 from Canada and 37 from UK). Residents, relatives of residents, or staff from 20 different LTC homes across England took part in the study. The recruitment strategy in Canada ensured that resident, family and staff participants were from multiple homes, however, no home-level identifying data were collected and thus the number of homes represented could not be reported. Demographic characteristics of the total sample were similar and are presented in Table 1 with separate characteristics in Appendix 2a-b. 72% of LTC residents, 78% of family and friends, and 94% of staff and clinicians were female. 83% of LTC residents, 97% of family and friends, and 76% of staff and clinicians were white. 50% of LTC residents had been diagnosed with dementia and 81% of family and friend interviewees reported that their relative or friend had dementia.

**Table 1:** Demographic characteristics of all study participants

		Residents (*n* = 18)	Family and friends (*n* = 32)	Staff and clinicians (*n* = 17)
Gender – n (%)	Female	13 (72)	25 (78)	16 (94)
Age (years)	Mean	82	64	40
	Range	69–99	40–85	24–60
Marital status – n (%)	Single	2 (11)	5 (16)	
	Married	3 (17)	21 (66)	
	Common-law	0 (0)	2 (6)	
	Separated	1 (5)	1 (3)	
	Divorced	3 (17)	1 (3)	
	Widowed	9 (50)	1 (3)	
	Other	0 (0)	1 (3)	
Employment status – n (%)	Employed	0 (0)	10 (30)	
	Retired	17 (94)	13 (41)	
	Unemployed	1 (6)	3 (9)	
	Other	0 (0)	6 (19)	
Race – n (%) Canadian participants *n* = 30	East/Southeast Asian	0 (0)	0 (0)	1 (25)
	White	5 (100)	21 (100)	2 (50)
	Other	0 (0)	0 (0)	1 (25)
Race – n (%) UK participants *n* = 37	Asian	0 (0)	0 (0)	2 (15)
	Mixed ethnicity	0 (0)	1 (10)	0 (0)
	White	10 (77)	10 (90)	11 (85)
	Other ethnic group	2 (15)	0 (0)	0 (0)
	Prefer not to disclose	1 (8)	0 (0)	0 (0)
Education - n (%)	Secondary/high school or less	7 (38)	1 (3)	1 (6)
	Degree	4 (21)	11 (34)	8 (47)
	Postgraduate	1 (7)	9 (28)	6 (35)
	Other	1 (7)	8 (25)	2 (12)
	Unknown	5 (27)	3 (10)	0 (0)
Diagnosis of dementia of resident - n (%)	Yes	9 (50)	26 (81)	
	No	9 (50)	6 (19)	
Length of time residing in care home of resident - n (%)	Less than 1 year	5 (28)	9 (28)	
	1–5 years	9 (50)	15 (47)	
	More than 5 years	4 (22)	7 (22)	
	Not disclosed	0 (0)	1 (3)	
Relationship to resident - n (%)	Spouse		7 (22)	
	Child		22 (69)	
	Sibling		1 (3)	
	Friend		1 (3)	
	Other		1 (3)	
Role - n (%)	Doctor/physician			3 (18)
	Recreation therapist / activity worker			3 (18)
	Personal support worker / care worker			2 (12)
	Clinical psychologist			1 (6)
	Care home manager / administrator			5 (28)
	Nurse			2 (12)
	Social worker			1 (6)
Years of experience - n (%)	Less than 5 years			5 (29)
	5–10 years			5 (29)
	More than 10 years			7 (42)
Working pattern - n (%)* (*n* = 13)	Full time			11 (85)
	Part time			2 (15)
Shift pattern - n (%)* (*n* = 13)	Days only			10 (80)
	Days and nights			3 (20)

Notes: * Includes only LTC home staff and not visiting professionals

Race categories are reported separately for each country as official categories for reporting race and ethnicity differ between Canada [28] and the UK [29]

Separate demographic tables for each country with their original categories can be found appendix 2a-b

Findings are grouped into four qualitative themes with 13 subthemes (Table 2). Each subtheme is structured through a narrative that explores its importance for social connection, followed by related barriers and facilitators. Quotes are prefixed by R for resident, F for family or friends, or S for LTC staff or clinicians.

**Table 2:** Qualitative themes and subthemes

Becoming familiar with life in the LTC home to support social connection	Counteracting the institutionalized feel of LTC homes
	Spending time in meaningful ways
	Increasing freedom of mobility
Physical and virtual access beyond the LTC home as strategies to maintain contact	Facilitating outings
	Providing support with technology
	Involving family and friends in LTC home life
Getting to know residents to deepen relationships	Using routine care and interactions as opportunities for social contact
	Using family and friend knowledge as a resource
	Fostering resident relationships
Person-centred approaches to build social connection	Considering health factors
	Accounting for adjustment and sociability Using communal spaces well
	Prioritizing psychosocial needs

### Becoming familiar with life in the LTC home to support social connection

Participants referred to ‘normality’ or ‘normal life’ to describe experiences that feel familiar and connect residents to their lives before LTC. This can be achieved when LTC residents do not feel institutionalized, are able to spend time in ways that feel meaningful to them and have freedom of mobility around the home.

### Counteracting the institutionalized feel of LTC homes

Living in an LTC home can create the sense of being institutionalized, which may distance LTC residents from feeling that they live a ‘normal’ life and therefore be detrimental to social connection. For example, when asked about her experience living in LTC, a resident described how rigid timetables can negatively impact her social life.

*“I didn’t like the regulation meal hours. And it’s like being at boarding school.” UK R1*.

Changing routine and creating variety, through spontaneous activities, for example, can facilitate social connection by counteracting the institutional feel of LTC homes.

*“We get loads of music on the television*,* but people are getting a bit fed up with stuff on the television […] But the other day*,* I sort of got up and I thought*,* oh*,* I just want to play them a little tune*,* and then the next thing you knew*,* the carers were all singing.” UK R2*.

Variety can also be introduced by providing LTC residents with a range of activities outside the home.

*“We plant flowers in the summertime. We go shopping sometimes […] It’s really enjoyable. Really.” Canada R1*.

Cultivating a culture of community and concern for others can also foster more personal relationships among residents and imitate the closer relationships they may have had prior to moving into LTC.

*“Sometimes when they see each other in the morning in the corridor*,* some of them give each other a hug. Ask if they*,* you know*,* ‘have you slept well?’” UK S1*.

This was reiterated by LTC residents, some of whom described closer relationships with each other from fostering a culture of togetherness.

*“I know and I understand their lives and it’s nice to talk about it […] It’s an understanding and togetherness*,* and from that*,* I suppose*,* some more than others I feel more drawn to.” UK R3*.

One staff member also described approaches for staff to make LTC homes feel more like a home.

*“It makes them happier that we don’t wear uniforms*,* the night staff at [the LTC home] wear pyjamas […] it does put down that barrier.” UK S2*.

### Spending time in meaningful ways

Spending time doing things they enjoyed was considered important for providing a sense of still living life and helping LTC residents to feel connected to and valued by others.

*“It’s the sense that […] they’re living and they’re doing and that they still matter.” UK S2*.

Family members observed their relatives can be under-stimulated if not offered engaging ways to spend time together.

*“The people just like*,* you know*,* sitting there staring at this TV and sometimes the TV wasn’t even playing and they’re just sitting there*,* and you know… There’s no stimuli.” Canada F1*.

This was reiterated by some staff members and was observed by both Canada and UK participants.

*“The TV’s blaring and nobody’s actually paying attention and*,* you know it’s … they might just say that ‘Oh*,* actually*,* there’s a group of people. Let’s put the TV on.’ But it’s not really meaningful.” UK S3*.

If LTC residents are under-stimulated, they may lack conversation topics which may limit the quality of social contact.

*“I think that there’s nothing to talk about […] one day is pretty much similar to the next and I think that just induces this sort of torpor.” UK F1*.

Some LTC residents said they avoided taking part in social activities if they have no interest in them.

*“I would never ever*,* ever play bingo in my life. Bloody stupid game. If ever you see me playing that*,* you know I’ve gone.” UK R4*.

On the other hand, some LTC residents were more engaged in meaningful activities they enjoyed. Establishing common ground and connecting residents to their shared generation and experiences, for example, provided them with material to bond over, enabled them to relate to one another and encouraged social interaction after the activity. For example, a staff member described the effects of reminiscence activities.

*“A lot of childhood stories end up being spoken about after an activity like that.” UK S4*.

Involving residents in the operations of LTC homes can connect them to past habits and give them a sense of purpose. This was considered to facilitate social connection, as some participants observed a link between enacting a sense of responsibility and increased engagement with peers.

*“We’ve got a nurse here*,* a retired nurse*,* and she folds bandages […] she’ll come round with us and do a ward round with us. We’ll walk around and check that everybody’s OK. […] It’s her truth.” UK S2*.

Both LTC residents and staff members expressed value in the consultation of residents in the operations of the LTC home, for example in resident meetings.

*“We have the resident meeting every month […] Most of the residents can say what they want to do. So it’s everybody’s collaboration*,* it’s not one person.” UK S4*.

LTC residents react positively when the LTC home actively takes their views and suggestions into account.

*“Very receptive. Yes*,* absolutely. If we make a suggestion*,* they do whatever they can to act on it.” UK R5*.

### Increasing freedom of mobility

If residents are enabled to move around the LTC home, this gives them choice and variety in who they spend time with.

*“We try and encourage that sort of neighbourliness […] they could quite easily have somebody round.” UK S5*.

Forged connections can be severed if LTC residents are separated, such as through policies which can separate people on different floors.

*“Cohorting is a barrier […] because [resident’s] friends are on the first floor and the third floor.” Canada F2*.

Conversely, freedom of mobility around the home can help to maintain relationships.

*“We have a few ladies that live on different floors so they will take the elevator and go see each other.” Canada S1*.

Some LTC residents described how the ability to visit each other enables them to carry out activities and spend more time together.

*“I go in this girl’s bedroom to help her read.” Canada R1*.

### Physical and virtual access beyond the LTC home as strategies to maintain contact

Opportunities to spend time outside the LTC home allows residents to maintain ties to family, friends and the community. This can be achieved through spontaneous or organized outings as well as outings with family or friends, supporting residents with their technology use, and encouraging the involvement of family and friends in LTC life.

### Facilitating outings

Leaving the LTC home enables residents to return to familiar environments and activities.

*“Sometimes they take me for dinner*,* I love it*,* go back to their homes because then I’m in a normal ordinary environment and that’s what I love.” UK R3*.

However, outings can be challenging to organize due to resource and staff shortages.

*“I like going out with [staff member]. But she can’t do that every day.” UK R7*.

Similarly, resident factors (e.g., health needs) can present additional barriers by impacting capacity to leave the LTC home. Many residents are unable to leave independently, and family and friends are often not equipped to manage resident needs themselves.

*“We have to take him out when there’s two of us*,* because I can’t physically manage him. His mobility is non-existent at the minute.” UK F2*.

However, successful approaches to bring the outside world into the LTC home were also described as opportunities for social connection.

*“The only reason she attends a community or religious meeting is because it’s in the actual facility*,* which I’m very grateful for […] I go with her and it’s become our new kind of routine where we go to church together.” Canada F1*.

### Providing support with technology

Although it does not replace in-person connection, virtual communication is a useful tool to facilitate social connection, particularly for LTC residents whose family and friends cannot regularly visit.

*“If families don’t have a choice*,* then this [video call] is the only way that they can get the social aspect and the emotional support.” Canada S1*.

When visitor restrictions were implemented during the COVID-19 pandemic, technology was the primary channel for LTC residents to maintain relationships and was described as a lifeline during this period.

*“Throughout COVID we were doing a lot of virtual visits so a lot of FaceTime visits with family and whatnot*,* helping to connect residents with their loved ones.” Canada S2*.

However, barriers were also discussed, whereby residents were not accustomed to the technology, which is typically not designed for older adults.

*“I can’t use them phones or anything*,* I’m hopeless*,* with technology.” UK R7*.

A family member explained the importance of support to help the resident overcome technological difficulties.

*“Part of the care plan was that […] there would be somebody there to give a hand with the phone because he often couldn’t hear it ringing anymore.” UK F2*.

However, staff levels do not always allow for technology support, and can make residents feel a burden so they hesitate to ask for help.

*“She requires assistance now to use her phone. And because of the shortness of staff at the home […] if she’ll ask them to dial my sisters or dial me… She feels like she’s a nuisance.” Canada F3*.

### Involving family and friends in LTC home life

Visits from family and friends enable LTC residents to maintain vital social connection.

*“[about receiving visits] I think it’s quite important*,* otherwise I’ll be quite isolated.” UK R4*.

For many, visits were seen as the main form of social connection, with observable impacts on residents.

*“She’s always cold and she doesn’t eat food or anything*,* and her daughter came one day and oh my goodness gracious. Her face just lit up.” Canada F4*.

Long-standing relationships provide a closeness often not achieved in newer LTC home relationships.

*“I don’t [think] there is anyone*,* apart from my friends who have known me all the years*,* that I feel I can share my feelings.” UK R3*.

However, it was acknowledged that visits can sometimes cause distress for some residents.

*“He sits there and holds my hand and… Sorry it’s going to make me cry… he says ‘I know you have to go. But I’d like if you can stay*,*’ And I say*,* ‘Dad*,* I can’t spend the night. I can’t stay here.’” Canada F5*.

In addition, some LTC residents may become overwhelmed or upset by visits, particularly with a larger group of visitors.

*“There were several people who came in to visit […] I think the number of people actually agitated her. I think her sense of movement*,* her sense of noise… A lot of things that you and I take for granted is so heightened that it makes her angry and upset.” Canada F6*.

LTC residents can benefit from social interactions with visitors of other residents. Family members noted the positive impact when they interacted with other residents. This was seen as particularly important for LTC residents who did not receive visits and experienced increased social isolation as a result.

*“If I talk to the lady that’s sitting next to my mom… You know*,* if I just chat her for a minute or ask her about her day*,* you can just see how much it impacts their health.” Canada F7*.

Bringing family and friends into the life of the LTC home can help residents engage in activities and increase social contact with others if they have a family member there to support them.

*“I’ve been trying to encourage her […] I would attend the different sessions*,* such as there was like*,* this clay thing where we were making a bowl. So I attended with her to help her to support her. And slowly she started to get more involved.” Canada F1*.

### Getting to know residents to deepen relationships

An improved understanding of LTC residents as individuals allows for closer and more trusting relationships, both from a resident-resident and staff-resident perspective. Staff members can use routine care and interactions as an opportunity to get to know the LTC residents, use the knowledge of family and friends to build a deeper understanding of them and foster resident relationships.

### Using routine care and interactions as opportunities for social contact

The staff-resident relationship was described as paramount to good social connection, and staff members can develop meaningful relationships with LTC residents during care.

*“If you go to someone for a task or you have a cup of tea or you got your meds or whatever*,* really trying to make the most of that interaction and establish some rapport with somebody.” UK S6*.

Although staff highlighted the importance of taking the time to get to know LTC residents, family members acknowledged that factors such as a heavy workload, turnover and staff shortages impact staff-resident relationships.

*“They are firefighting. There’s nothing you can do about it*,* but they’re going from breakfast to lunch to dinner*,* they’re doing a lot*,* and they’re cleaning him up […] they are doing marvellous job in the circumstances*,* but you can’t expect much more.” UK F3*.

With this in mind, care can be used as an opportunity to build rapport.

*“Getting somebody up and dressed is a connection and an activity*,* letting them choose which clothes they want to wear […] That’s a connection.” UK S2*.

LTC residents find value in staff members making conversation and building an understanding of them during care, helping them feel more comfortable.

*“He would*,* you know*,* joke with different staff or get along better in terms of feeling more at ease with someone changing and doing all that stuff.” Canada F8*.

Joking around and laughing together was also described as conducive to closer staff-resident relationships.

*“Aye*,* banter […] So I usually say*,* ‘is he coming in today? Well*,* tell him I want a kiss when he comes in’*,* things like that.” UK R8*.

Comfortable staff-resident relationships develop naturally over time for many, so staff turnover can impede staff-resident relationships.

*“It’s nice having the regulars. Good for them and it’s good for us.” UK R3*.

### Using family and friend knowledge as a resource

Family and friends hold a wealth of knowledge about residents which LTC homes can use to tailor care plans, improve social connection, and ease the process of settling in.

*“It was about speaking to the son and saying could you fill out this ‘getting to know me’ paperwork just so that*,* you know*,* this lady’s got a lot of anxiety*,* just about: is there something we’re missing? Did she like to listen to the piano when she was younger before she got her illness? What is going to relax her?” UK S7*.

Information provided by family and friends can be translated in practical ways to get to know LTC residents, including posters or noticeboards, which provide content for staff, other residents and visitors to engage residents.

*“[A poster] does tell the staff that don’t know her something about her*,* so that if they choose to talk to her it gives them something to say to her that maybe she could engage them with.” Canada F7*.

This knowledge can also be used as a resource for LTC homes to support resident relationships and facilitate potential friendships.

*“We do as much as we can to find out about the person before they come in […] And then the wellbeing team really act on pairing them up with somebody […] and then they sit together and stuff like that. So that that that works really*,* really well. But you’ve got to know about the person.” UK S2*.

### Fostering resident relationships

Resident relationships are important for many and can help LTC residents feel part of the LTC community.

*“He knows one or two people. They sit in*,* which is excellent*,* on the landing […] He can feel all part of the community here.” UK F3*.

However, many LTC residents reported feeling set apart from other residents and asserted they have nothing in common with them.

*“I don’t think they’re my type of people.” UK R6*.

This reduced motivation to socialize, whereas others lacked motivation to form new friendships.

*“How can you make new relationships at 80? What are you offering?” UK R1*.

Some staff members perceived that residents are brought together by their shared circumstances including living in an LTC home together.

*“People get frailer*,* they probably can’t communicate as well. And but that seems to be all accepted. That seems to be just part of it. We’re all in the same sort of boat type of a feeling.” UK S2*.

However, some LTC residents did not agree.

*“We’ve all had to give up our homes […] So we’ve all been through a traumatic experience. Yeah*,* but it’s not an experience that draws us together*,* it’s weird.” UK R1*.

Despite this contrast, opportunities to understand each other’s backgrounds and challenges may help LTC residents develop more empathetic relationships and can be facilitated by activities. One resident described how her perception of another resident changed once she developed a deeper understanding of her, leading her to empathize with behaviour she had previously found challenging.

*“We were talking about where people come from*,* and their backgrounds*,* and all she said to us was this ‘I’m 98*,* I was brought up in Germany*,* Hitler*,* don’t ask me anymore.’ […] now I appreciate sometimes why she shouts and behaves the way she does.” UK R3*.

Another consideration when matching LTC residents is hobbies and interests to set up potential friendships.

*“This woman really enjoyed gardening her entire life and this gentleman really enjoys gardening and so if we invite them both to a gardening group or*,* you know*,* maybe perhaps that would kind of begin a friendship.” UK S8*.

### Person-centred approaches to build social connection

Resident-level factors such as complex health and psychosocial needs, and sociability and adjustment to life in LTC, significantly impact social connection. LTC homes can build an awareness of individual needs and abilities, use communal spaces effectively, and tailor care, interventions, and activities accordingly.

### Considering health factors

Many LTC residents have complex health needs relating to their cognitive, physical, sensory and mental health. Sensory impairment is linked to social isolation, whereby those with poor eyesight or hearing may not be aware of or able to interact with the people around them.

*“She just doesn’t really know if there’s somebody there or not [due to vision impairment]. So*,* it’s kind of lonely unless somebody actually comes up and talks to her.” Canada F9*.

Sensory impairment can also impact capacity to take part in activities, leading some to miss out on social opportunities.

*“I can’t see well enough for bingo […] My eyesight is getting worse.” Canada R2*.

Cognitive impairment can impact capacity to join conversations and reduce social interactions. It can also hinder the development of resident relationships, as memory loss can make forged connections short-lived.

*“She suffers from very bad dementia. So she couldn’t recognize me.” UK R9*.

Other health factors, such as aphasia or speech impediments, cause communication difficulties for LTC residents.

*“We sit at meals […] She’s a nice*,* nice person that has a speech impediment. So it’s hard to understand her all the time.” Canada R3*.

Without support, LTC residents with physical health needs such as low mobility can spend more time alone in their rooms.

*“They can’t go to the TV room if they want. They can’t go to a neighbor’s room and have a chat if they want.” Canada F10*.

Depression can impact motivation to socialize or go out reducing social engagement and increasing isolation for some.

*“I think the most challenging is for people who are depressed […] because their mood […] makes it more difficult for them to feel connected or to want to connect.” UK S6*.

Some family members expressed frustration at a lack of activities suitable for specific health needs, as it prevents LTC residents from taking part and experiencing their social benefits.

*“I thought for God’s sake*,* don’t you people get it? She’s blind […] they can’t individualize anything*,* I guess is what I mean. Like that doesn’t make sense to give a blind lady a painting kit*,* you know?” Canada F11*.

Activities can be individualized to health needs. For example, activities that do not centre around conversation allow those with communication or cognitive impairments to form bonds in other ways. However, although individualized activities can lead to improved engagement, staff members acknowledge the challenge in individualizing for LTC residents experiencing varying levels of cognitive impairment.

*“We can’t generalize the activities most of the times here*,* because of their levels of dementia and their social interactions are totally different. So… it’s hard to have the common ground for all of them.” UK S4*.

Some LTC residents reported strained relationships with those with dementia, and that cognitive impairment creates a barrier to socializing, particularly during mealtimes. Conversely, some LTC residents may feel increased kinship towards others in similar circumstances. For example, cognitively intact LTC residents can sit together during mealtimes or activities.

*“For some residents it’s a lot harder for them to communicate and to do things independently. And if you’ve sat at the table with them*,* you might have no conversation […] these ladies that are at your table*,* then you can actually talk about stuff because they’re quite sociable too.” Canada F12*.

Similarly, LTC residents with dementia can attend tailored activities, such as sensory activities, helping them feel more comfortable and encouraging social interactions. However, others described the positive impact of mixing cognitively intact residents with those with dementia to provide support and engaging social interactions.

*“To have two people who find it a little bit hard to communicate with each other trying to communicate*,* versus one person who finds it pretty easy and one person who finds a little bit hard*,* that communication would obviously be a little more fluid.” UK S8*.

Staff members observed the importance of non-verbal interactions for severely cognitively impaired LTC residents who may be post-verbal:

*“She will make eye contact with certain individuals […] her eyes are like glued to that person and she waves her hand and she*,* you know*,* things like that. So the relationships are still active and building even with the residents that are post-verbal.” UK S8*.

LTC residents facing barriers due to health needs can be supported by a staff member or volunteer.

*“She went every Friday for the bingo*,* and she can’t see. But one of the volunteers sat with her and she thoroughly enjoyed that.” Canada F3*.

LTC residents spend a lot of time around others and learn about each other in the process, which some participants cite as a resource. Some report helping other LTC residents with health needs and developing a bond with them this way, and express joy at both helping and being helped. This can include practical support….

*“She looks forward to mealtimes at the facility and because of her requirement now she needs to have someone assist her with feeding […] lady friends have been her support. They indicate to her what is on her plate.” Canada F3*.

…as well as emotional support.

*“She was moved with the lady that had was bedridden all the time and speechless […] she tried to nurse her. She would go to her bedside and take her hand*,* and she was… She would be caring.” Canada F13*.

#### Accounting for adjustment and sociability

The ease with which residents experience life in an LTC home is influenced by previous circumstances and personal characteristics. Those who were previously socially isolated may find a busy LTC home environment overwhelming, whilst others thrive with increased social activity. Introverted LTC residents may have different needs when engaging with others.

*“There are people who just generally in life*,* you know*,* are not club joiners or people who*,* you know*,* prefer to stay isolated or to themselves. And so*,* we also have to figure out how to meet their needs.” Canada S2*.

Some do not seek relationships, and feel connected in their own company, content spending extended periods of time in their rooms engaged in solitary activities that are still meaningful to them.

*“I watch BBC4*,* because they have a lot of very interesting history and human studies that have been done […] So I don’t feel lonely.” UK R9*.

However, a solitary experience in an LTC home is not always a choice, and some may spend time on their own because they are unwell, under-stimulated, or need extra encouragement to socialize. It may be helpful for LTC residents with a tendency to withdraw to be encouraged to take part in social activities.

*“You ask someone if they want to do it and they actually are going to say no most of the time. So maybe some people would need someone to say*,* ‘well*,* you might come along and try it.’” UK R3*.

Some LTC residents expressed reluctance to accept life in LTC and resist opportunities for social engagement. It can take time to become accustomed to this new way of living and feel comfortable around others. Equally, it takes time for staff to work out how best to engage individual LTC residents. Awareness of individual needs is crucial to understand why some LTC residents do not actively socialize.

*“My mum doesn’t eat her lunch with the other residents and that’s not because she’s antisocial […] it’s because she removes her denture before she eats her lunch and she wouldn’t want to do that in front of other people.” UK F4*.

### Using communal spaces well

Communal spaces provide opportunities to be around others. Even if LTC residents are not actively engaging with each other, the physical presence of others can be comforting for some.

*“Just being able to sit back and hear other people and not feel alone I think is huge for many.” Canada F8*.

Some LTC residents reported increased social interactions with other residents as a result of being in communal spaces.

*“I can’t walk […] but somebody can take me out in the hallway. Some of the ladies will come over and say*,* ‘how are you?’” Canada R4*.

However, the physical presence of others alone does not always alleviate feelings of loneliness or provide comfort for all LTC residents.

*“Residents are constantly surrounded with others and there is an assumption that they aren’t sort of deeply lonely or deeply alone.” Canada S3*.

In addition, communal spaces can be stress-inducing environments for LTC residents.

*“Some days I’m all wearisome and it’s because some of the residents*,* the way they go on arguing with each other and things like that*,* you know*,* I can’t cope with all of that.” UK R2*.

Communal spaces alone were therefore not always conducive to positive social connection. Active encouragement of social engagement, such as during activities or events, can maximize the social benefits of these spaces for LTC residents who do not feel comforted solely by the presence of others, and can help bring them together.

*“When there’s kind of big stuff happening*,* Jubilee time*,* Queen’s funeral… It was just like ‘No*,* this is a really big deal we’re all going to get together and take this in together.” UK F5*.

The outside spaces of LTC homes can also be used to target individual interests and encourage social engagement with other LTC residents.

*“And then we all go outside together and put them around the garden*,* and then it comes back to reminiscing about*,* like a lot of people are interested in birds*,* they know which bird is which.” UK S1*.

#### Prioritizing psychosocial needs

Social connection is facilitated when care actively focuses on psychosocial as well as physical needs. Psychosocial care is important for health and wellbeing and participants express value in it being prioritized as such. This was evidenced during the COVID-19 pandemic, when staff-resident interactions were reduced, and emotional support compromised as a result.

*“She would say*,* you know*,* ‘people just pop in my room to give me my food and then they’re off again and everyone’s in masks.’ So honestly*,* I think that was the biggest thing and that was a bit heart-breaking […] They were doing everything right*,* but that was really hard.” UK F5*.

An emphasis on psychosocial needs can be supported through the culture that is created within the home.

*“[A culture] where the staff are encouraged and allowed to spend that time*,* where that’s seen as important. And where there’s a culture around communicating compassionately with people […] You can reassure someone who’s hugely distressed*,* you can get to the bottom of their unmet needs and understand who they are as a person.” UK S6*.

Small opportunities to make LTC residents feel valued can improve their psychosocial wellbeing. Family members highlighted physical touch as a way to achieve this.

*“Touch their hand*,* touch their shoulder*,* let them know that they’re still human and that there’s still a person […] all these things would help I think maintain those social connections.” Canada F5*.

If staff build awareness of individual psychosocial needs, they can care for LTC residents in ways that transcend physical care.

*“The relationships with staff that I’ve observed is that they’re incredibly fond of you*,* very caring*,* and they look out for you. The other day*,* they actually took action to stop you being visited by someone who you don’t like.” UK F6*.

## Discussion

This qualitative study is, to our knowledge, the first to consider multiple key collaborator perspectives on the barriers and facilitators to social connection in LTC homes and identified four themes. *Becoming familiar with life in the LTC home to support social connection* is enabled by counteracting the institutionalized feel of LTC homes, residents spending time meaningfully and having freedom of mobility around the home. *Physical and virtual access beyond the LTC home as strategies to maintain contact* is facilitated by outings, support with technology use and involving family and friends in LTC life, through visits and personal support during activities. *Getting to know residents to deepen relationships* is important for staff-resident and resident-resident relationships and can be achieved by using routine care and interactions as opportunities for social contact and using family and friend knowledge as a resource. *Person-centred approaches to build social connection* are important to help LTC residents feel valued. It is influenced by factors related to health, adjustment and individual sociability, and psychosocial needs, and can be facilitated through using communal spaces effectively and tailoring care and activities. We aimed to investigate multiple perspectives to create an overview of individual-level and home-level factors impacting social connection in LTC homes.

Our findings support previous research that opportunities to find meaning in LTC homes are associated with both individual- and home-level factors [30], and restoring a sense of home and familiarity in LTC residents is antithetical to the perception of being institutionalized [31]. We found strategies to achieve this that add to previous research, such as using spontaneous activities to create variety in the LTC home, and encouraging a culture of community and care amongst residents. Activities previously enjoyed as community-dwellers have been linked to improved adjustment and increased social engagement [32]. Personalized activities were found to improve social connection in our previous study which analyzed qualitative interviews from one of the research sites in this study, focusing on how different components of activities in LTC homes affect social connection [33]. The current study builds on previous research by considering barriers and facilitators to social connection beyond care home activities. Opportunities to share memories and develop an understanding of peers can improve socialization and reduce loneliness in LTC residents who are both cognitively intact [34] and cognitively impaired [35]. Although our findings support such activities as facilitators of social connection, we identified complex health needs as a significant barrier to participation. In addition, our findings provide further evidence of the division between residents with varying levels of cognitive impairment [8, 36]. We identify specific challenges LTC homes face in managing this, namely in tailoring activities and interventions designed to improve social connection to a cohort of LTC residents with varying needs.

A lack of autonomy in who LTC residents choose to spend time with can impede their sense of control and negatively impact social engagement [37]. LTC homes must balance shelter and care with freedom and autonomy [38]. LTC residents in the present study valued the freedom to move around the LTC home and maintain existing relationships, demonstrating such mobility as a pathway to increased autonomy. However, safety concerns related to cognitive impairment and physical dependence limits autonomy for many LTC residents [30].

Our study complements literature establishing family involvement as a crucial facilitator of social connection [37], but family and friends in our study added that there are potential detrimental impacts of visits such as distress and agitation, particularly for those with cognitive impairment. However, literature suggests visits are linked to reduced, rather than increased, agitation in LTC residents with dementia [39]. In our study, detrimental impacts were noted to be an occasional consequence of larger groups visiting, consistent with previous recommendations that visits may prove most beneficial when visitors come in smaller numbers [37].

LTC residents expressed the positive impact of staff-resident relationships that incorporate trust and comfort, and valued joviality and laughter with staff. Evidence from interventions links humor therapy to decreased feelings of loneliness [40] and perceived loneliness [41]. Fun and friendships are important aspects of making LTC residents feel ‘at home’ [42], as is staff members knowing LTC residents [43], and LTC residents feeling known and valued as individuals [31]. Reciprocity, acquaintance with personal preferences and a caring attitude contributes towards LTC residents’ perception of close staff-resident relationships [44]. Our findings about the positive impact of more personal staff-resident relationships and interventions that can help strengthen these relationships, such as during routine care or with the help of family and friend knowledge, present further evidence of the importance of and need for interventions to improve psychosocial wellbeing. We identified two macro-level factors impacting social connection that appeared throughout our analysis. Firstly, the negative impact of the COVID-19 pandemic was consistently perceived as a barrier to social connection. This is consistent with studies linking COVID-19 restrictions to increased loneliness [45] and impaired communication with staff members [46]. The regular references to the negative impact of COVID-19 restrictions serve to demonstrate social engagement and support as prerequisites to feelings of social connectedness. A second aspect running throughout our findings is the detrimental impact of low staffing levels and high staff turnover on the social connection of LTC residents. Although staff members often champion an LTC culture that values psychosocial wellbeing, LTC resident and family and friend perspectives lay bare the reality that LTC homes may be unable to facilitate close relationships [44], prioritize psychosocial needs and implement supportive policies [47] if they lack the sufficient resources. Consistent with these findings, the present study highlights the negative impact of insufficient resources, underlining time staff spend getting to know LTC residents and building personal relationships with them as a crucial pathway to good social connection.

### Strengths and limitations

Our study presents data from multiple key collaborator perspectives, allowing for an overview of barriers and facilitators to social connection that incorporates different views and priorities across LTC residents, staff and clinicians, and family and friends. Further, we collected our data from LTC homes in both Canada and the UK and found similar experiences across both countries, which may increase the cross-cultural transferability of our findings. However, despite efforts to sample purposively, our sample lacked ethnic diversity, with most residents being white, meaning that our results may not represent the experiences of LTC residents from minority ethnic or other underrepresented backgrounds. This is an important consideration as there is evidence to suggest disparities in the social experiences of LTC residents across different ethnic and cultural backgrounds [48]. Our convenience sample of LTC residents is likely to have meant that participants were more sociable than LTC residents who did not participate, so we have less awareness of the experiences of more socially isolated residents. In addition, our criteria that LTC residents had mental capacity to provide informed consent may also have introduced bias into the sample, as LTC residents without adequate mental capacity may be more likely to experience lower levels of social connection. Our criteria that family members were only eligible to participate if they visited the LTC resident at least monthly limits the representation of the experiences of family members who less frequently visit their LTC dwelling relative, though we ensured that we heard from staff about the experiences of residents without family and spoke to residents who did not have family who visited regularly. In addition, we did not collect data about the range of sizes, structures, and policies of individual LTC homes, which may factor into the levels of social connection for residents in different homes. Lastly, staff members who participated may have been from higher quality homes, been more interested in their role, or have wanted to portray their homes in a positive light.

### Conclusions and implications

This qualitative study identifies individual- and home-level factors, as well as macro-level issues such as COVID-19, that influence social connection in LTC homes. Our findings reveal key aspects of life in LTC that may prevent or enable residents from engaging with others, developing trusting relationships, expanding their social networks or feeling socially connected. We used multiple perspectives and experiences to improve our understanding of mechanisms underlying social connection in LTC and offer examples of care practices which can help to overcome them. Our findings identify areas that require further input in interventions and policy, namely in the prioritization of psychosocial needs and individualized support as pathways to improved social connection. For example, interventions such as addressing vision and hearing loss, using technology to communicate, and communicating non-verbally have been proposed to be beneficial [49], all of which appear to be supported by our qualitative findings so should be made systematically available for all residents. In addition, all key participant groups noted that cognitive impairment can contribute to the social divide between LTC residents and indicated additional challenges in tailoring care and interventions to respective needs and abilities, so further research may therefore consider how to optimize care for LTC residents with different levels of cognitive impairment living alongside each other. Our findings can be addressed in care policies, activity programming and staff selection, as well as psychosocial interventions to build and maintain social connection in LTC homes. Such policies and interventions could be integrated into staff training in communication and care, and may hold potential to improve health and quality of life for LTC residents.

## Electronic Supplementary Material

Below is the link to the electronic supplementary material.

Supplementary Material 1

Supplementary Material 2

Supplementary Material 3

## Data Availability

We are not sharing data in order to protect study participant privacy as per the terms of our ethical approval, as it may contain information that may make participants identifiable.
