# Supplementary material for: Social connection in long-term care homes: a qualitative study of barriers and facilitators
Source: BMC Geriatr. 2024 Oct 22;24:857. doi: 10.1186/s12877-024-05454-8 (PMC11494782; doi:10.1186/s12877-024-05454-8)
Supplement: Supplementary file 1 — Supplementary Material 1 [file 12877_2024_5454_MOESM1_ESM.docx]

**Appendix 1a.** Semi-structured interview guide (LTC resident version)

**Topic guide – LTC resident**

**Social connection in long-term care residents**

**Introduction**

Thank you for taking part in this interview. We would like to know what is important for people in terms of social connection, meaning the experience of social contact, relationships and activities for people living in care homes. I will ask about what sort of social connection takes place in care homes, and what may be missed in terms of social connection. We will also show you some different ways that researchers currently use to measure social connection and ask for your views on these as we want to improve how we measure this so that we and others can conduct research and improve services in the future.

To make sure I don’t miss anything, I will record our conversation on a digital recorder and I may make some notes as you speak.

I am interested in hearing about your thoughts and experiences so I’d like you to say as much as you can about the topics we discuss, [and I would like to hear from both of you].

**START DIGITAL RECORDER**

Social Connection before moving to LTC home

**First, I would like to find out about how life was for you before you moved to the long-term care home.**

**Was socialising and spending time with others, like family or friends, a big part of your life?**

Additional prompts:

- Were you the type of person who enjoyed spending time with others or did you prefer more solitary activities?

**What social activities were important to you?**

Additional prompts:

- Did you enjoy visiting family and friends or having them to visit?
- Did you engage in cultural activities like going to the cinema, concerts, theatre?
- Did you enjoy activities at home such as reading or watching television?
- Were volunteering, community activities or religious groups important?
- What about sports or exercise groups?
- Did you get involved with any support groups?

**Were you socially isolated or saw people less often than you wanted to?**

Additional prompts:

- Were there family and friends nearby that could visit?
- Did people visit often?
- Did you speak to others on the telephone or via other means of communication?
- Who initiated these contacts?
- Were you limited by health issues or practical issues (like driving)?
- **Were you lonely?**

Preparing to move to LTC home

**Were there activities which you were concerned that you would miss out on once you moved to the care home?**

**Were there new activities which you hoped you would be able to access in the care home (things that would be better)?**

**Did you expect your social contact to improve or worsen on moving to care home?**

Additional prompts:

- Would you see others as often as usual?
- Would it matter that the people you would have contact with might not initially be known to you?
- Would having contact with staff members matter?
- Were you worried about how much you would be able or allowed to participate in activities?

Since moving to LTC home

**Now I would like to ask about your experience of the [*NAME*] care home.**

**Can you tell me about your social life in the LTC home?**

Additional prompts:

- Do you have family and friends come to visit in the home? Has the pattern of visits changed over time?
- Do you leave the home to meet family and friends or go to events?
- Can you tell me how you feel you spend time socializing with other residents?
- Can you tell me about your relationships with staff?
- Did you prefer solitary activities, like reading or watching television in your room?

**Has it been like this since you first moved in - or has it changed over time?**

Additional prompts:

- If it has changed over time, how and why has it changed?

**What activities are important to you within the care home?**

Additional prompts:

- Art or music?
- Storytelling?
- Exercise groups?
- Other group activities?

**Do you consider social connection overall to be important for your wellbeing?**

**What would you consider to be good social connection in a care home?**

**Are there things you think influence building or maintaining social connection in a care home?**

Additional prompts:

- Things that get in the way?
- Things that help?
- Staff time?
- Resident’s health, such as issues of mobility, cognitive impairment or vision and hearing?
- Things about the home, like how the building is designed or policies that are put in place?

**Do you feel there are people in the care home who know you well?**

Additional prompts:

- Any close relationships with members of staff?
- Any close relationships with other residents?
- Do you feel like your likes/dislikes/personal preferences are known to any other people and are valued in the care home?

Ways of assessing social connection

**When asking about anyone's social connection, we typically want to ask the person themselves. But this is not always possible in care homes so we sometimes ask family or staff. From your experience, what do you think would be the best way to get information about residents' social connection?**

- Why is that?

**Now I would like to ask you about ways in which researchers have previously asked about social connection. I will show you some questions from other scale and I would like your views on these.**

Closing questions

**We are coming towards the end of our conversation now. Is there anything else you would like to add?**

*If a need is identified, signpost to the appropriate service.*

Thank you for speaking to me.

**END OF INTERVIEW - STOP DIGITAL RECORDER**

**Appendix 1b.** Semi-structured interview guide (family and friend version)

**Topic guide – family/friend**

**Social connection in long-term care residents**

**Introduction**

Thank you for taking part in this interview. We would like to know what is important for people in terms of social connection, meaning the experience of social contact, relationships and activities for people living in care homes. I will ask about what sort of social connection takes place in care homes, and what may be missed in terms of social connection. We will also show you some different ways that researchers currently use to measure social connection and ask for your views on these as we want to improve how we measure this so that we and others can conduct research and improve services in the future.

To make sure I don’t miss anything, I will record our conversation on a digital recorder and I may make some notes as you speak.

I am interested in hearing about your thoughts and experiences so I’d like you to say as much as you can about the topics we discuss, [and I would like to hear from both of you].

**START DIGITAL RECORDER**

Social Connection before moving to LTC home

**First, I would like to find out about how life was for your relative before they moved to the long-term care home.**

**Was socialising and spending time with others, like family or friends, a big part of their life?**

Additional prompts:

- Were they the type of person who enjoyed spending time with others or did they prefer more solitary activities?

**What social activities were important to them?**

Additional prompts:

- Did they enjoy visiting family and friends or having them to visit?
- Did they engage in cultural activities like going to the cinema, concerts, theatre?
- Did they enjoy activities at home such as reading or watching television?
- Were volunteering, community activities or religious groups important?
- What about sports or exercise groups?
- Did they get involved with any support groups?

**Were they socially isolated or saw people less often than they wanted to?**

Additional prompts:

- Were there family and friends nearby that could visit?
- Did people visit often?
- Did they speak to others on the telephone or via other means of communication?
- Who initiated these contacts?
- Were they limited by health issues or practical issues (like driving)?
- **Did you feel that they were lonely?**

Preparing to move to LTC home

**Were there activities which you were concerned that they would miss out on once they moved to the care home?**

**Were there new activities which you hoped they would be able to access in the care home (things that would be better)?**

**Did you expect their social contact to improve or worsen on moving to care home?**

Additional prompts:

- Would they see others as often as usual?
- Would it matter that the people they would have contact with might not initially be known to them?
- Would having contact with staff members matter?
- Were they worried about how much they would be able or allowed to participate in activities?

Since moving to LTC home

**Now I would like to ask about your experience of the [*NAME*] care home.**

**Can you tell me about your relative's social life in the LTC home?**

Additional prompts:

- Do they have family and friends come to visit in the home? Has the pattern of visits changed over time?
- Do they leave the home to meet family and friends or go to events?
- Can you tell me how you feel they spend time socializing with other residents?
- Can you tell me about their relationships with staff?
- Did they prefer solitary activities, like reading or watching television in their room?

**Has it been like this since they first moved in - or has it changed over time?**

Additional prompts:

- If it has changed over time, how and why has it changed?

**What activities are important to them within the care home?**

Additional prompts:

- Art or music?
- Storytelling?
- Exercise groups?
- Other group activities?

**Do you consider social connection overall to be important for their wellbeing?**

**What would you consider to be good social connection in a care home?**

**Are there things you think influence building or maintaining social connection in a care home?**

Additional prompts:

- Things that get in the way?
- Things that help?
- Staff time?
- Resident’s health, such as issues of mobility, cognitive impairment or vision and hearing?
- Things about the home, like how the building is designed or policies that are put in place?

**Do you feel there are people in the care home who know your relative well?**

Additional prompts:

- Any close relationships with members of staff?
- Any close relationships with other residents?
- Do you feel like your / your relative’s likes/dislikes/personal preferences are known to any other people and are valued in the care home?

Ways of assessing social connection

**When asking about anyone's social connection, we typically want to ask the person themselves. But this is not always possible in care homes so we sometimes ask family or staff. From your experience, what do you think would be the best way to get information about residents' social connection?**

- Why is that?

**Now I would like to ask you about ways in which researchers have previously asked about social connection. I will show you some questions from other scale and I would like your views on these.**

Closing questions

**We are coming towards the end of our conversation now. Is there anything else you would like to add?**

*If a need is identified, signpost to the appropriate service.*

Thank you for speaking to me.

**END OF INTERVIEW - STOP DIGITAL RECORDER**

**Appendix 1c.** Semi-structured interview guide (staff member and clinician version)

**Topic guide – Researchers / LTC staff and clinicians**

**Social connection in long-term care residents – Aim 2**

**Introduction**

Thank you for taking part in this interview. We would like to know what is important for people in terms of social connection, meaning their experience of social contact, relationships and activities for people living in care homes. I will ask about what sort of social connection takes place in care homes, and what may be missed in terms of social connection. We will also show you some different ways that researchers currently use to measure social connection and ask for your views on these as we want to improve how we measure this so that we and others can conduct research and improve services in the future.

To make sure I don’t miss anything, I will record our conversation on a digital recorder and I may make some notes as you speak.

I am interested in hearing about your thoughts and experiences so I’d like you to say as much as you can about the topics we discuss, [*and I would like to hear from everyone*].

**START RECORDING**

1.0 Social connection in care homes

I would like to ask about your experience of social connection in long-term care homes.

*[If no experience of visiting/working of care home, move to ‘2.0 General’ section below.]*

**What activities are available within the care home you work in and/or visit? And have these been helpful for improving social connection?**

**How is social contact with others organised?**

Additional prompts:

- Visits from family or friends
- Visits from other people
- Activities with other residents (either organized by the staff or spontaneous)
- Hybrid events (e.g. guest singers coming in and family members can join them)

2.0 General

**Do you consider social connection overall to be important for care home residents’ wellbeing?**

**Does it have any other effects for care home residents or staff?**

Additional prompts:

- enjoyment
- residents getting to know each other
- reducing boredom
- reducing agitation
- creativity of residents
- ability to sing or paint for example when cannot do other things
- keep people awake so they sleep better

**Are there any negative effects?**

Additional prompts:

- arguments
- people not liking each other
- people being left out of group

**Do you think that social connection changes for residents when they enter a care home compared to life before?**

Additional prompts:

- If so, what changes?
- What have caused these?

**After moving in to a care home, do you think that residents’ social connection changes over time?**

Additional prompts:

- If so, what changes?
- What have caused these?

**What would you consider to be a good social connection in a care home?**

3.0 Ways of assessing social connection

**When asking about a long term care resident’s social connection, we could ask the resident themselves, their family relative and the paid staff.**

**Who do you feel would be best placed to provide information about social connection?**

**Why is that?**

**If the resident has cognitive impairment or dementia, does it affect this?**

**We could ask objective questions about the amount of time people spend doing sociable activities or their subjective experience of those activities e.g. satisfaction, engagement, or loneliness.**

**Which of these is important?**

**Why is that?**

**If the resident has cognitive impairment or dementia, does it affect this?**

**Now I would like to ask you about ways in which researchers have previously asked about social connection. I will show you some questions from other scale and I would like your views on these.**

**I would like to know whether these questions are relevant to residents with and without dementia.**

**I would also like to know what you think about the response options and whether you think they are relevant to**

4.0 Closing questions

**We are coming towards the end of our conversation now. Is there anything else you would like to add?**

Thank you for speaking to me.

**END OF INTERVIEW - STOP RECORDING**
