# Supplementary material for: Social connection in long-term care homes: a qualitative study of barriers and facilitators
Source: BMC Geriatr. 2024 Oct 22;24:857. doi: 10.1186/s12877-024-05454-8 (PMC11494782; doi:10.1186/s12877-024-05454-8)
Supplement: Supplementary file 2 — Supplementary Material 2 [file 12877_2024_5454_MOESM2_ESM.docx]

**Appendix 2a.** Demographic characteristics of Canadian study participants

|  |  | **Residents**  **(n = 5)** | **Family and friends (n = 21)** | **Staff**  **(n = 4)** |
| --- | --- | --- | --- | --- |
| Gender – n (%) | Female | 4 (80) | 17 (81) | 4 (100) |
|  | Male | 1 (20) | 4 (19) | 0 (0) |
|  | Prefer not to disclose | 0 (0) | 0 (0) | 0 (0) |
|  | Other | 0 (0) | 0 (0) | 0 (0) |
| Age (years) | Mean | 81 | 63 | 39 |
|  | Range | 69-99 | 40-80 | 25-53 |
| Marital status –  n (%) | Single | 0 (0) | 3 (14) |  |
|  | Married | 1 (20) | 14 (66) |  |
|  | Common-law | 0 (0) | 1 (5) |  |
|  | Separated | 1 (20) | 1 (5) |  |
|  | Divorced | 1 (20) | 1 (5) |  |
|  | Widowed | 2 (40) | 1 (5) |  |
|  | Other | 0 (0) | 0 (0) |  |
| Employment status – n (%) | Employed | 0 (0) | 10 (31) |  |
|  | Retired | 4 (80) | 13 (41) |  |
|  | Unemployed | 1 (20) | 3 (9) |  |
|  | Other | 0 (0) | 6 (18) |  |
| Race – n (%)  Canadian participants | Black | 0 (0) | 0 (0) | 0 (0) |
|  | East/Southeast Asian | 0 (0) | 0 (0) | 1 (25) |
|  | Indigenous | 0 (0) | 0 (0) | 0 (0) |
|  | Latino | 0 (0) | 0 (0) | 0 (0) |
|  | Middle Eastern | 0 (0) | 0 (0) | 0 (0) |
|  | South Asian | 0 (0) | 0 (0) | 0 (0) |
|  | White | 5 (100) | 21 (100) | 2 (50) |
|  | Other | 0 (0) | 0 (0) | 1 (25) |
|  | Prefer not to disclose | 0 (0) | 0 (0) | 0 (0) |
| Education – n (%)  Canadian participants | Less than high school/secondary school | 2 (40) | 0 (0) | 0 (0) |
|  | High school/secondary school | 0 (0) | 0 (0) | 0 (0) |
|  | College, CEGEP, non-university diploma | 1 (20) | 7 (33) | 0 (0) |
|  | Undergraduate/bachelor degree | 1 (20) | 9 (43) | 3 (75) |
|  | Master’s degree or higher | 1 (20) | 5 (24) | 1 (25) |
|  | Other | 0 (0) | 0 (0) | 0 (0) |
| Diagnosis of dementia of resident - n (%) | Yes | 2 (40) | 17 (81) |  |
|  | No | 3 (60) | 4 (19) |  |
|  | Unknown | 0 (0) | 0 (0) |  |
| Length of time residing in care home of resident - n (%) | Less than 1 year | 0 (0) | 5 (24) |  |
|  | 1-5 years | 3 (60) | 10 (48) |  |
|  | More than 5 years | 2 (40) | 6 (28) |  |
| Relationship to resident - n (%) | Spouse |  | 4 (19) |  |
|  | Child |  | 17 (81) |  |
|  | Son/daughter-in-law |  | 0 (0) |  |
|  | Sibling |  | 0 (0) |  |
|  | Friend |  | 0 (0) |  |
|  | Other |  | 0 (0) |  |
| Role - n (%) | Academic researcher  Care home manager/administrator  Clinical psychologist  Doctor/physician  Nurse  Occupational therapist  Personal support worker/care worker  Recreation therapist/activity worker  Social worker  Other | |  | 0 (0) |
|  |  |  |  | 0 (0) |
|  |  |  |  | 0 (0) |
|  |  |  |  | 0 (0) |
|  |  |  |  | 1 (25) |
|  |  |  |  | 0 (0) |
|  |  |  |  | 0 (0) |
|  |  |  |  | 1 (25) |
|  |  |  |  | 1 (25) |
|  |  |  |  | 1 (25) |
| Years of experience - n (%) | Less than 5 years |  |  | 1 (25) |
|  | 5-10 years |  |  | 3 (75) |
| Working pattern –  n (%) | Full time |  |  | 4 (100) |
| Shift pattern –  n (%) | Days only |  |  | 3 (75) |
|  | Days and nights |  |  | 1 (25) |

**Appendix 2b.** Demographic characteristics of UK study participants

|  |  | **Residents**  **(n = 13)** | **Family and friends**  **(n = 11)** | **Staff**  **(n = 13)** |
| --- | --- | --- | --- | --- |
| Gender – n (%) | Female | 9 (69) | 8 (73) | 12 (92) |
|  | Male | 4 (31) | 3 (27) | 1 (8) |
|  | Prefer not to disclose | 0 (0) | 0 (0) | 0 (0) |
|  | Other | 0 (0) | 0 (0) | 0 (0) |
| Age (years) | Mean | 82 | 68 | 40 |
|  | Range | 74-91 | 55-85 | 24-60 |
| Marital status –  n (%) | Single | 2 (15) | 2 (18) |  |
|  | Married | 2 (15) | 7 (64) |  |
|  | Common-law | 0 (0) | 1 (9) |  |
|  | Separated | 0 (0) | 0 (0) |  |
|  | Divorced | 2 (15) | 0 (0) |  |
|  | Widowed | 7 (55) | 0 (0) |  |
|  | Other | 0 (0) | 1 (9) |  |
| Employment status – n (%) | Employed | 0 (0) | 4 (36) |  |
|  | Retired | 13 (100) | 5 (46) |  |
|  | Unemployed | 0 (0) | 1 (9) |  |
|  | Other | 0 (0) | 1 (9) |  |
| Race – n (%)  UK participants | Asian or Asian British | 0 (0) | 0 (0) | 2 (15) |
|  | Black, African, Caribbean or Black British | 0 (0) | 0 (0) | 0 (0) |
|  | Mixed ethnicity | 0 (0) | 1 (10) | 0 (0) |
|  | White | 10 (77) | 10 (90) | 11 (85) |
|  | Other | 2 (15) | 0 (0) | 0 (0) |
|  | Prefer not to disclose | 1 (8) | 0 (0) | 0 (0) |
| Education - n (%)  UK participants | Primary | 0 (0) | 0 (0) | 0 (0) |
|  | Lower secondary | 2 (15) | 0 (0) | 1 (9) |
|  | Higher secondary | 3 (23) | 1 (9) | 0 (0) |
|  | Degree | 3 (23) | 2 (18) | 5 (38) |
|  | Postgraduate | 0 (0) | 4 (36) | 5 (38) |
|  | Other | 0 (0) | 1 (10) | 2 (15) |
|  | Unknown | 5 (38) | 3 (27) | 0 (0) |
| Diagnosis of dementia of resident - n (%) | Yes | 7 (54) | 9 (82) |  |
|  | No | 6 (46) | 2 (18) |  |
|  | Unknown | 0 (0) | 0 (0) |  |
| Length of time residing in care home of resident - n (%) | Less than 1 year | 5 (38) | 4 (36) |  |
|  | 1-5 years | 6 (46) | 5 (46) |  |
|  | More than 5 years | 2 (16) | 1 (9) |  |
|  | Not disclosed | 0 (0) | 1 (9) |  |
| Relationship to resident - n (%) | Spouse |  | 3 (28) |  |
|  | Child |  | 5 (45) |  |
|  | Son/daughter-in-law |  | 0 (0) |  |
|  | Sibling |  | 1 (9) |  |
|  | Friend |  | 1 (9) |  |
|  | Other |  | 1 (9) |  |
| Role - n (%) | Academic researcher  Care home manager/administrator  Clinical psychologist  Doctor/physician  Nurse  Occupational therapist  Personal support worker/care worker  Recreation therapist/activity worker  Social worker  Other | |  | 0 (0) |
|  |  |  |  | 5 (39) |
|  |  |  |  | 1 (8) |
|  |  |  |  | 2 (15) |
|  |  |  |  | 1 (8) |
|  |  |  |  | 0 (0) |
|  |  |  |  | 2 (15) |
|  |  |  |  | 2 (15) |
|  |  |  |  | 0 (0) |
|  |  |  |  | 0 (0) |
| Years of experience - n (%) | Less than 5 years |  |  | 4 (31) |
|  | 5-10 years |  |  | 4 (31) |
|  | More than 10 years |  |  | 5 (38) |
| Working pattern - n (%)* n=9 | Full time |  |  | 7 (78) |
|  | Part time |  |  | 2 (22) |
| Shift pattern –  n (%)* n=9 | Days only |  |  | 7 (78) |
|  | Days and nights |  |  | 2 (22) |

* Includes LTC staff only and not visiting professionals
