# Supplementary material for: Social connection in long-term care homes: a qualitative study of barriers and facilitators
Source: BMC Geriatr. 2024 Oct 22;24:857. doi: 10.1186/s12877-024-05454-8 (PMC11494782; doi:10.1186/s12877-024-05454-8)
Supplement: Supplementary file 3 — Supplementary Material 3 [file 12877_2024_5454_MOESM3_ESM.docx]

**Appendix 3.** Consolidated criteria for reporting qualitative studies (COREQ): 32-item checklist

| **No.** | **Item** | **Description** | **Page No.** |
| --- | --- | --- | --- |
| **Domain 1: Research team and reflexivity** | | | |
| Personal characteristics | | | |
| 1. | Interviewer | Individual interviews, dyadic interviews for some residents and family members, and LTC staff, and a focus group were semi-structured and lead by one researcher (AS a consultant psychiatrist, ES a research analyst, HC a research assistant, JB an epidemiologist, MM a postgraduate student, and two research nurses from one NHS trust, DD and LJ), and co-facilitated by a second researcher in some cases. | Page 6 |
| 2. | Researcher credentials | Author 1: BA, MSc  Author 2: BSc, MSc, PhD  Author 3: BSc, NPT, PhD  Author 4: BSc, MSc  Author 5: MBChB, MD, FRCPsych  Author 6: RN, PhD, FAAN, FCAHS, FCAN  Author 7: BSc, MBBS, MSc, PhD, MRCPsych | N/A |
| 3. | Occupation | The research team consisted of 1) a research assistant with postgraduate psychology training, 2) an epidemiologist, 3) a research associate, 4) a PhD candidate, 5 &7) two consultant old age psychiatrists, and 6) a professor in interventions and care delivery models for persons with cognitive impairment. | N/A |
| 4. | Gender | Authors 1 to 6 are female. Author 7 is male. | N/A |
| 5. | Experience and training | Authors 1 and 4 had postgraduate-level experience in research methods and additional academic training in qualitative methods. Author 3 had doctoral level experience in research methods. Authors 2, 5, 6 and 7 are experienced researchers in the field of social connection in care homes and dementia. | N/A |
| Relationship with participants | | | |
| 6. | Relationship established | In the UK, LTC residents, family and friends, and staff were recruited from LTC home liaison teams in three NHS trusts: Camden and Islington NHS Foundation Trust, Oxford Health NHS Foundation Trust and Northumbria Healthcare NHS Foundation Trust, and from a London LTC home organization. Staff were also recruited through dissemination of information about the study to the ENRICH national care home research network (<https://enrich.nihr.ac.uk/>). In Canada, participants were recruited through networks and organizations representing LTC residents, families and staff across Ontario, Canada as well as through individually operated and chain LTC homes. | Page 6 |
| 7. | Participant knowledge of the interviewer | Authors 1 and 2 had no previous relationship with any of the participants. Author 7 had no previous relationship with any of the LTC residents, family and friends, or LTC staff, but had a professional relationship with two clinicians interviewed in a focus group. | N/A |
| 8. | Interviewer characteristics | Interviews were conducted by a member of the research team (AS, HC, or MM, or two research nurses in the UK, and JB and ES Canada), and co-facilitated by a second researcher in some instances. | Page 6 |
| **Domain 2: Study design** | | | |
| Theoretical framework | | | |
| 9. | Methodological orientation and theory | We used Braun and Clarke’s thematic analysis with deductive reasoning to identify themes in line with prior research and existing theory. | Page 7 |
| Participant selection | | | |
| 10. | Sampling | We used purposive sampling and recruited participants across Canada and the UK, including LTC residents, family and friends of LTC residents, and LTC staff and clinicians, to explore multiple key collaborator perspectives. | Page 5 |
| 11. | Method of approach | Study posters were sent out widely to advertise the study. LTC home managers helped to identify eligible residents and staff members. Interested participants were given the opportunity to ask questions and time to decide whether to take part before a meeting online or in person was arranged to obtain informed consent and conduct the interview. | Page 5 |
| 12. | Sample size | We interviewed 67 participants: 18 LTC residents, 17 staff members, and 32 family or friends of residents. | Page 8 |
| 13. | Non-participation | In Canada, no participants refused participation or dropped out during the study. In the UK, three residents refused participation, one resident was deemed to lack capacity to consent to participation. No carers or staff refused to participate. No participants dropped out during the study. | N/A |
| Setting | | | |
| 14. | Setting of data collection | In the UK, all LTC resident interviews were conducted in-person in LTC homes, and staff and clinician interviews were conducted virtually using the video-calling platform Microsoft Teams. Family and friend interviews were conducted both in-person and virtually. In Canada, interviews were conducted either in-person in LTC homes (for some resident and family interviews) and virtually for all other participants. | Page 6 |
| 15. | Presence of non-participants | Some LTC residents were interviewed in the presence of a family member. | N/A |
| 16. | Description of sample | 72% of LTC residents, 78% of family and friends, and 94% of staff and clinicians were female. 83% of LTC residents, 97% of family and friends, and 76% of staff and clinicians were white. 50% of LTC residents had been diagnosed with dementia and 81% of family and friend interviewees reported that their relative or friend had dementia. | Page 8, Table 1, appendix 2a-b |
| Data collection | | | |
| 17. | Interview guide | We developed interview guides for each participant type based on previous study findings and our research questions. Interview questions explored aspects of social connection since moving into an LTC care home, including the quality of residents’ social life, how it has changed over time, and what influences building or maintaining social connection in this environment. | Page 6, appendix 1a-c |
| 18. | Repeat interviews | No repeat interviews were carried out. | N/A |
| 19. | Audio/visual recording | Interviews were recorded using Microsoft Teams or an audio-recorder and transcribed verbatim. | Page 6 |
| 20. | Field notes | No field notes were made. | N/A |
| 21. | Duration | Interviews took 30-60 minutes. | Page 6 |
| 22. | Data saturation | We stopped recruiting and interviewing participants once we judged that we had reached data saturation for each participant group, meaning that no new themes emerged during our data analysis. | Pages 6-7 |
| 23. | Transcripts returned | We did not return transcripts to the participants. | N/A |
| **Domain 3: Analysis and findings** | | | |
| Data analysis | | | |
| 24. | Number of data coders | All members of the research team coded data, with one researcher per interview transcript. | Page 6 |
| 25. | Description of the coding tree | We selected a subsample of five interview transcripts representing different views and experiences, including interviews from each key collaborator group, to develop a coding framework [23]. The codebook was guided by the data and pre-defined research objectives, and described both objective and experienced aspects of living in LTC homes. All five members of the research team (AS, HC, JB, ML, ND) each reviewed the selected interview transcripts to extract new codes. | Page 7 |
| 26. | Derivation of themes | Theme identification was driven by existing codes and used deductive reasoning to identify themes in line with prior research and existing theory. | Page 8 |
| 27. | Software | Dedoose v9.0.17 | Page 7 |
| 28. | Participant checking | We did not conduct participant checking. | n/a |
| Reporting | | | |
| 29. | Quotations presented | Participant quotes are included throughout the results to illustrate each theme. Quotes were anonymized, prefixed (R for resident; S for LTC staff and clinicians; F for family and friends) and numbered by the order in which they appear. | Pages 10-27 |
| 30. | Data and findings consistent | Themes were established based on experiences and views shared amongst several participants within the three participant groups in order to ensure consistency in the data and findings. | Pages 10-27 |
| 31. | Clarity of major themes | Major themes were clearly presented in the results, with a summary of each major theme provided before the dissection of subthemes. | Pages 10-27, Table 2 |
| 32. | Clarity of minor themes | Subthemes explored nuances in the views and experiences amongst different participant groups. | Pages 10-27 |
